# Intravascular adhesion and recruitment of neutrophils in response to CXCL1 depends on their TRPC6 channels

**DOI:** 10.1007/s00109-020-01872-4

**Published:** 2020-01-16

**Authors:** Otto Lindemann, Jan Rossaint, Karolina Najder, Sandra Schimmelpfennig, Verena Hofschröer, Mike Wälte, Benedikt Fels, Hans Oberleithner, Alexander Zarbock, Albrecht Schwab

**Affiliations:** 1 Institute of Physiology II, Westfälische Wilhelms-Universität, Münster, Germany; 2 Department of Anaesthesiology, Intensive Care and Pain Medicine, University Hospital Münster, Münster, Germany; 3 Institute of Cell Dynamics and Imaging, Westfälische Wilhelms-Universität, Münster, Germany

**Keywords:** TRPC6 channel, Neutrophil recruitment, CXCL1, CXCR2

## Abstract

**Abstract:**

Here we report a novel role for TRPC6, a member of the transient receptor potential (TRPC) channel family, in the CXCL1-dependent recruitment of murine neutrophil granulocytes. Representing a central element of the innate immune system, neutrophils are recruited from the blood stream to a site of inflammation. The recruitment process follows a well-defined sequence of events including adhesion to the blood vessel walls, migration, and chemotaxis to reach the inflammatory focus. A common feature of the underlying signaling pathways is the utilization of Ca^2+^ ions as intracellular second messengers. However, the required Ca^2+^ influx channels are not yet fully characterized. We used WT and TRPC6^−/−^ neutrophils for in vitro and TRPC6^−/−^ chimeric mice (WT mice with WT or TRPC6^−/−^ bone marrow cells) for in vivo studies. After renal ischemia and reperfusion injury, TRPC6^−/−^ chimeric mice had an attenuated TRPC6^−/−^ neutrophil recruitment and a better outcome as judged from the reduced increase in the plasma creatinine concentration. In the cremaster model CXCL1-induced neutrophil adhesion, arrest and transmigration were also decreased in chimeric mice with TRPC6^−/−^ neutrophils. Using atomic force microscopy and microfluidics, we could attribute the recruitment defect of TRPC6^−/−^ neutrophils to the impact of the channel on adhesion to endothelial cells. Mechanistically, TRPC6^−/−^ neutrophils exhibited lower Ca^2+^ transients during the initial adhesion leading to diminished Rap1 and β_2_ integrin activation and thereby reduced ICAM-1 binding. In summary, our study reveals that TRPC6 channels in neutrophils are crucial signaling modules in their recruitment from the blood stream in response to CXCL1.

**Key point:**

Neutrophil TRPC6 channels are crucial for CXCL1-triggered activation of integrins during the initial steps of neutrophil recruitment.

## Introduction

Neutrophil recruitment occurs in a precisely orchestrated sequence of events (reviewed in Filippi [1]). In its early phase, the binding of endothelial selectins to P-selectin glycoprotein ligand 1 (PSGL1) triggers an intracellular signaling cascade, resulting in inside-out signaling via PI3K (phosphatidylinositide-3-kinase) and PLC (phospholipase-C). This causes the activation of β_2_-integrins, such as LFA1 (integrin α_L_β_2_), to an extended conformation with intermediate binding affinity for its ligands [2]. The binding of extended LFA1 to intercellular adhesion molecule 1 (ICAM-1) induces neutrophil slow rolling on endothelial cells. Thereby, neutrophils are brought into close proximity to the surface of endothelial cells so that they can respond to inflammatory chemoattractants, which are trapped in the glycocalyx of inflamed endothelial cells [3]. IL-8 and CXCL1 induce the extension of LFA1 to its high-affinity conformation in human [4] and murine [5] neutrophils which leads to their firm arrest on endothelial cells. Macrophage-1 antigen (Mac1), the second β_2_-integrin which is abundantly expressed on neutrophils, facilitates intravascular crawling of neutrophils from the site of adhesion to the site of emigration through the endothelium into the perivascular tissue [6].

It is well established that multiple steps of the neutrophil recruitment cascade are linked to intracellular Ca^2+^ signaling. Thus, the activation of G protein coupled CXC-motif chemoattractant receptor 2 (CXCR2) by its ligand CXCL1 involves PLC activation and the generation of inositol 1,4,5-trisphosphate (IP_3_) and diacylglycerol (DAG) [7]. IP_3_ triggers the Ca^2+^ release from intracellular stores and subsequent store-operated Ca^2+^ entry via Orai1 channels [8]. DAG can directly activate members of the transient receptor potential (TRP) channel family, like TRPC6, leading to Ca^2+^ influx from the extracellular space [9]. Several Ca^2+^-permeable channels such as Orai1 channels [8, 10] as well as TRPC1 [11], TRPC6 [12, 13], TRPM2 [14], and TRPV4 channels [15] in neutrophils have been shown to be involved in their recruitment (reviewed in [16, 17]). However, the elucidation of the underlying mechanisms by which these Ca^2+^ influx channels regulate neutrophil recruitment is complicated by the multitude of chemoattractants. While there seems to be a coupling between TRPC6 channels and CXCR2, data concerning the link between TRPM2 channels and fMLP-triggered signaling are discussed controversially [16]. It is further complicated by the fact that Ca^2+^ signaling in endothelial (or epithelial) cells has to be considered, too. Indeed, the abovementioned channels are also expressed in endothelial cells [18] and several members of the TRP channel family in endothelial (or epithelial) cells have also been shown to regulate neutrophil recruitment [19–21] (reviewed in [16, 22]).

Based on their link to CXCR2 signaling and the fact that CXCR2 is involved in intravascular arrest of neutrophils [23], a role for TRPC6 channels in the intravascular recruitment steps appears likely. Since TRPC6 channels largely mediate the CXCR2-induced rise of the intracellular Ca^2+^ concentration [12], they could trigger the activation of the Ca^2+^- and DAG-activated guanine nucleotide exchange factor CalDAG-GEF1 [24]. CalDAG-GEF1 in turn is an important module of the signaling cascade underlying integrin activation [25, 26]. Mutations in CalDAG-GEF1 cause leukocyte adhesion deficiency syndrome (LAD III) [27]. In this study, we tested whether TRPC6 channels are central elements of the signaling cascade underlying CXCR2-mediated neutrophil recruitment. We combined intravital microscopy, single-cell force spectroscopy with atomic force microscopy, Ca^2+^ imaging, and microfluidic flow chamber assays to investigate the role of TRPC6 channels in murine neutrophils for their recruitment in renal ischemia-reperfusion and cremaster models as well as in in vitro assays.

## Methods

### Animals

All animal experiments were approved by the local authorities (Landesamt für Natur, Umwelt und Verbraucherschutz Nordrhein-Westfalen). Mixed chimeric mice were generated by performing bone marrow transplantation as previously described [28]. Bone marrow cells isolated from WT and TRPC6^−/−^ donor mice were injected intravenously into lethally irradiated C57BL/6J WT-recipient mice. The resulting chimeric mice are designated as WT/WT mice (WT mice with WT bone marrow cells) or WT/TRPC6^−/−^ mice (WT mice with TRPC6^−/−^ bone marrow cells). In vitro experiments were performed with bone marrow–derived neutrophils isolated either from C57BL/6J WT mice or their TRPC6^−/−^ littermates.

### Ischemia-reperfusion injury model

The ischemia-reperfusion injury (IRI) model has been described previously [29]. Ischemia was induced by bilateral renal pedicle clamping for 32 min. Kidneys were inspected for immediate color change, indicating successful clamping. After clamp removal, kidneys were checked for a change in color within 3 min to ensure reperfusion. In animals subjected to sham operation, the surgical procedure was identical except that no clamps were applied. Incisions were closed in two layers and animals were allowed to recover. After 24 h, the mice were euthanized, blood samples were taken by heart puncture, the kidneys were flushed by perfusion through the left ventricle, and both kidneys were harvested to determine the number of neutrophils in the kidney. Serum creatinine levels were determined by using a creatinine assay (Diazyme, Poway, USA) according to the manufacturer’s protocol. PMN recruitment into the kidneys was analyzed by flow cytometry after 24 h [29].

### Intravital microscopy

Intravital microscopy was performed as described before [30]. Mice were anesthetized using injection of 125 mg/kg ketamine hydrochloride and 12.5 mg/kg xylazine intraperitoneal. For intravital microscopy (IVM), the cremaster muscle of anesthetized mice was exteriorized and inflammation was induced by superfusion with the respective chemoattractant (CXCL1 5 nM, fMLP 100 μM) for 1 h. Postcapillary venules with a diameter between 20 and 40 μm were investigated. Leukocyte arrest was analyzed by transillumination intravital microscopy, whereas leukocyte extravasation was investigated by near infrared reflected light oblique transillumination (RLOT) microscopy [31]. IVM was performed on an upright microscope (Axioskop; Zeiss, Göttingen, Germany) equipped with a 40 × 0.75 NA saline immersion objective and a digital camera (Sensicam QE, Corporation, Romulus, USA). Blood flow centerline velocity was measured using a dual photodiode sensor system (CircuSoft Instrumentation, Hockessin, USA) in order to ensure comparable microvascular hemodynamics in between the groups [32]. Blood flow centerline velocity was measured using a dual photodiode sensor and digital online cross-correlation program (CircuSoft Instrumentation, Hockessin, USA). Centerline velocities were converted to mean blood flow velocities by multiplying with an empirical factor of 0.625 [33]. Wall shear rates (*γ*_W_) were estimated as 4.9 (8v_b_/d), where v_b_ is the mean blood flow velocity, d is the diameter of the vessel, and 4.9 is a median empirical correction factor obtained from velocity profiles measured in microvessels in vivo [34]. Image analysis was performed using ImageJ (version 1.48) and AxioVision (Zeiss, Göttingen, Germany) software. Emigrated cells were analyzed in an area of 100 μm blood vessel length × 75 μm to each side of a vessel (representing 1.5 × 10^4^ μm^2^ tissue area). Leukocyte arrest was determined before and every minute after intravenous injection of 600 ng CXCL1 [23]. Arrest was defined as leukocyte adhesion longer than 30 s and expressed as cells per surface area.

### Measurement of the cytosolic Ca^2+^ concentration

The measurement of the cytosolic Ca^2+^ concentration ([Ca^2+^]_i_) was performed in μ-slide I chambers (ibidi, Martinsried, Germany). To mimic the physiological conditions during capturing of neutrophils, μ-slides were coated with 3.5 μg/ml E-selectin (R&D Systems, Minneapolis, USA) overnight. Unspecific binding sites were blocked (1% fat-free milk in PBS) for 1 h. When indicated, this was followed by 2 h coating with 1.43 μg/ml CXCL1 in blocking solution. Afterwards, the slides were washed with 2 ml of Ringer solution. Neutrophils from overnight cultures were pelleted (4 °C, 1000 rpm, 10 min), resuspended in RPMI 1640 containing 25 mM HEPES, and preincubated with 3 μM of the Ca^2+^ dye Fura-2-AM (Calbiochem, Gibbstown, USA) at 4 °C. After 20 min, cells were pelleted (4 °C, 1000 rpm, 10 min) and resuspended in Ringer solution. During the measurement, a laminar flow of 5 dyn/cm^2^ was maintained in the slide using a tubing pump (Masterflex C/L 10–60 rpm, 12 VDC, Cole-Parmer, Vernon Hills, USA). Cells were brought into the flow of this system, and [Ca^2+^]_i_ was measured during the initial rolling phase. Experiments were carried out at room temperature, and images were acquired in 500-ms intervals. At the end of each experiment using slides coated with E-selectin and CXCL1, the measurements were calibrated by applying 1 μM ionomycin (MP Biomedicals, Solon, USA) containing Ringer solution with 5 mM EGTA or 5 mM Ca^2+^. [Ca^2+^]_i_ was calculated as described before [35]. The area under the curve was calculated as integral for the first 5 s after initial contact to the selectin-coated surface (normalized to base levels of [Ca^2+^]_i_ the cells reached after a few minutes). When slides were only coated with E-selectin, a calibration was not possible because the vast majority of neutrophils did not adhere firmly and rapidly rolled out of the visual field. In this case, we only analyzed background-corrected ratio values of the emissions after exciting at 340 nm and 380 nm. Further experimental details were described earlier [12].

### Single-cell force spectroscopy

Single-cell force spectroscopy experiments were performed by using atomic force microscopy (AFM) (CellHesion® 200, JPK, Berlin, Germany) as described before [36]. All experiments were analyzed using JPK Data Processing (software version 4.2.50). Arrow TL-1 tipless cantilevers (NanoAndMore GmbH, Wetzlar, Germany) were incubated prior to experiments for 30 min in Cell-Tak (Corning, NY, USA) to make the AFM cantilever sticky for neutrophils. bEnd5 endothelial cells were grown in a monolayer in glass bottom dishes using flexiPERM cell culture inserts (Sarstedt, Nümbrecht, Germany). Twenty-four hours prior to the experiment, bEnd5 cells were stimulated with 5 nM TNFα (PeproTech, Hamburg, Germany). The surrounding glass bottom was coated with 10% FCS in PBS overnight and washed twice with water. Afterwards, cell culture inserts were removed and the whole dish was washed twice with Ringer solution. Neutrophils were seeded besides the endothelial cells, and the front part (apex) of the Cell-Tak-coated cantilever was brought into contact with a single PMN for 5 s using a maximal loading force of 1 nN in order to attach the PMN firmly to the AFM cantilever. Measurements were performed in Ringer solution (control) or Ringer solution containing 1.43 μg/ml CXCL1 at 37 °C. The process of attaching neutrophils to cantilevers was highly standardized and optically controlled so that the neutrophils were always at the same position of the cantilever and had the same morphology.

Force-distance curves were obtained by probing bEnd5 cells with the neutrophil-carrying cantilever using 1 nN maximal loading force (Fig. 4a). Positioning of neutrophils above and lowering onto endothelial cells was optically controlled so that they were always placed in the middle between cell nucleus and cell-cell junctions of endothelial cells. To mimic the rapid time course of the formation of adhesive bonds between neutrophils and endothelial cells during rolling and adhesion, the contact time was set to 1 s. Further specific AFM parameters were as follows: average AFM cantilever spring constant: 0.02–0.025 N/m; average deflection sensitivity: 90 nm/V; *z*-length (pull-off distance): 50 μm; cantilever velocity during approach/retraction: 5 μm/s; sampling rate: 205 Hz. In average, 30 force-distance curves were performed per one individual neutrophil on ten individual endothelial cells. Adhesion forces were determined by analyzing the individual maximum pulling forces.

### Rap1 Tat-fusion mutants

The G12V mutation was introduced into the Rho family small GTP binding protein Rap1A (RAP1A00000) via the QuikChange mutagenesis kit (StrataGene, La Jolla, USA). This mutation induces a constitutively active phenotype (CA). The Tat-proteins from HIV-1 have been shown to be easily taken up by different cell types [37]. The Tat-fusion mutants have been generated as described previously [38].

### Microflow chamber assays

The effect of Rap1 Tat-fusion mutants was analyzed in a previously described microflow chamber system [39]. Rectangular glass capillaries (20 µm × 200 μm) were filled either with E-selectin (2.5 μg/mL, R&D Systems) and ICAM-1 (2 μg/mL, R&D Systems) alone or in combination with CXCL1 (0.07 μg/ml) for 2 h and then blocked for 1 h using casein (Pierce Chemicals, Dallas, TX, USA). One side of the chamber was connected to a PE 50 tubing (Becton Dickinson) and used to control the wall shear stress in the capillary. The other side of the chamber was inserted into a syringe filled with heparinized whole blood. Whole blood samples from WT or TRPC6^−/−^ mice were incubated with TAT-fusion mutants (1 μM, 37 °C, 30 min) and adherent neutrophils were analyzed by microscopy. One representative field of view was recorded for 1 min using an SW40/0.75 objective and a digital camera (Sensicam QE; Cooke Corporation, Romulus, USA).

Alternatively, we used μ-slides that were coated o/n with 3.5 μg/ml E-selectin and blocked for 1 h with 1% fat free milk in PBS. 500,000 neutrophils (WT or TRPC6^−/−^) were suspended in 1 ml HEPES-buffered Ringer’s solution supplemented with different concentrations of CXCL1 (0.03–30 μg/ml) or fMLP (10 nmol/L–5 μmol/L). When indicated, 3 μg/ml CXCL1 were supplemented with 5 μmol/l larixyl acetate. This suspension was flown at 5 dyn/cm^2^ through the μ-slides using a tubing pump (Masterflex C/L 10–60 rpm, 12 VDC, Cole-Parmer, Vernon Hills, USA). After 2 min, the adherent cells were fixed with 3.5% paraformaldehyde for 30 min. Adherent neutrophils were quantified in three visual fields that were placed at the identical distance from the entrance into the microchannel.

### Active Rap1 pull-down assay

Active Rap1 Pull-Down and Detection Kit was purchased from Thermo Scientific (Pierce Biotechnology, Rockford, USA). A GST-fusion protein of the Rap1-binding domain is coupled to a glutathione agarose resin to specifically pull down active Rap1, which is analyzed by Western blot. For the Pull-Down reaction neutrophils from overnight cultures were pelleted (4 °C, 1000 rpm, 10 min), washed once with RPMI 1640 containing 25 mM HEPES and stimulated for 30 s with 1.43 μg/ml CXCL1. Afterwards, cells were pelleted and lysed following the manufacturer’s protocol. For each Pull-Down reaction, 400 μg total protein was used and the direct flow-through after the pull-down containing the remaining total protein was used as additional loading control in Western blots.

### ICAM-1 binding assay

Integrin binding to LFA-1 was analyzed as described before [29, 40]. Neutrophils from overnight cultures were incubated with CD11b (clone M1/70; 10 μg/ml) to avoid Mac-1 binding to ICAM-1. In the presence of ICAM-1/Fc (20 μg/ml) and allophycocyanin-conjugated IgG (Fc specific) cells were stimulated with CXCL1 (0.1 μg/ml) or left unstimulated in control experiments. Control experiments had shown that ICAM-1 binding is abolished when neutrophils are preincubated with blocking LFA-1 antibodies [40]. ICAM-1 binding was analyzed using flow cytometry.

### Statistics

Data are presented as means ± SEM. All data were tested for normality and evaluated for statistically significant differences (*p* < 0.05) by means of Student’s *t* test or Mann-Whitney *U* test. Multiple comparison was tested with ANOVA and Tukey post hoc test. Data outliers were detected with Grubbs or Nalimov tests.

## Results

### Renal damage after ischemia-reperfusion is attenuated in TRPC6^−/−^mice

**Fig. 1:**
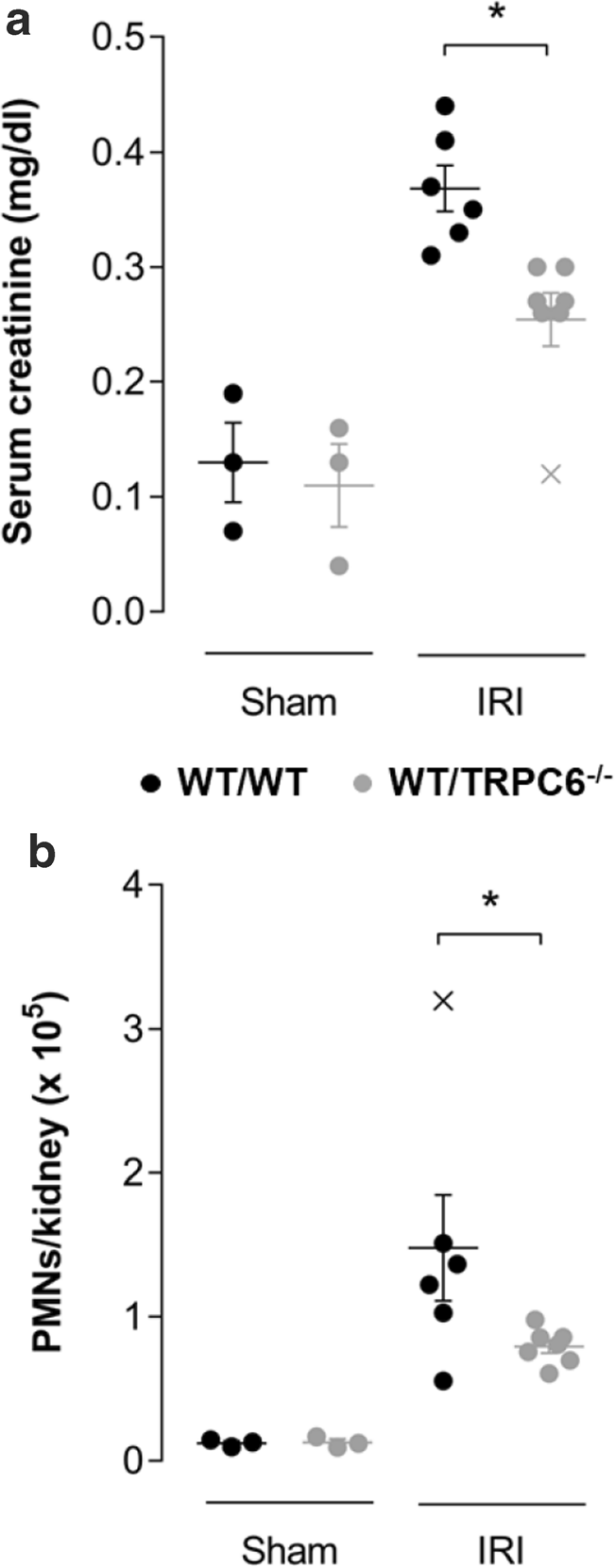
Neutrophil recruitment and renal damage after ischemia and reperfusion is reduced in WT/TRPC6^−/−^ mice. Serum creatinine (**a**) and renal neutrophils (**b**) after ischemia/reperfusion injury (sham: *n* = 3, IRI: *n* ≥ 6 mice/group). Values are reported as mean values ± SEM. *p < 0.05. Data points represented by a cross (X) were identified as outliers and not considered for statistical analysis

To investigate the pathophysiological relevance of the TRPC6 channels in neutrophils, we induced renal ischemia-reperfusion injury (IRI) in WT/WT and WT/TRPC6^−/−^ chimeric mice. After 24 h of reperfusion, the serum creatinine levels were determined to assess renal function, and the number of neutrophils in the kidneys was analyzed as a measure of renal inflammation. Serum creatinine levels and neutrophils in the kidney were similar in sham operated WT/WT and WT/TRPC6^−/−^ chimeric animals. Serum creatinine increased in WT/WT mice after renal ischemia. In WT/TRPC6^−/−^ mice the increase was ~ 30% lower (Fig. 1a). We observed a similar difference between WT/WT and WT/TRPC6^−/−^ mice with respect to the renal neutrophil count. While the number of neutrophils in the kidneys of WT/WT mice strongly increased, the neutrophil count was ~ 50% lower in WT/TRPC6^−/−^ mice (Fig. 1b). The improved outcome of the renal ischemia-reperfusion injury of WT/TRPC6^−/−^ chimeric mice is consistent with the idea that TRPC6 channels contribute to the recruitment of neutrophils into the kidneys so that the deletion of TRPC6 channels in neutrophils is protective under this condition.

### Loss of TRPC6^−/−^ channels reduces adhesion and transmigration of neutrophils in vivo

**Fig. 2:**
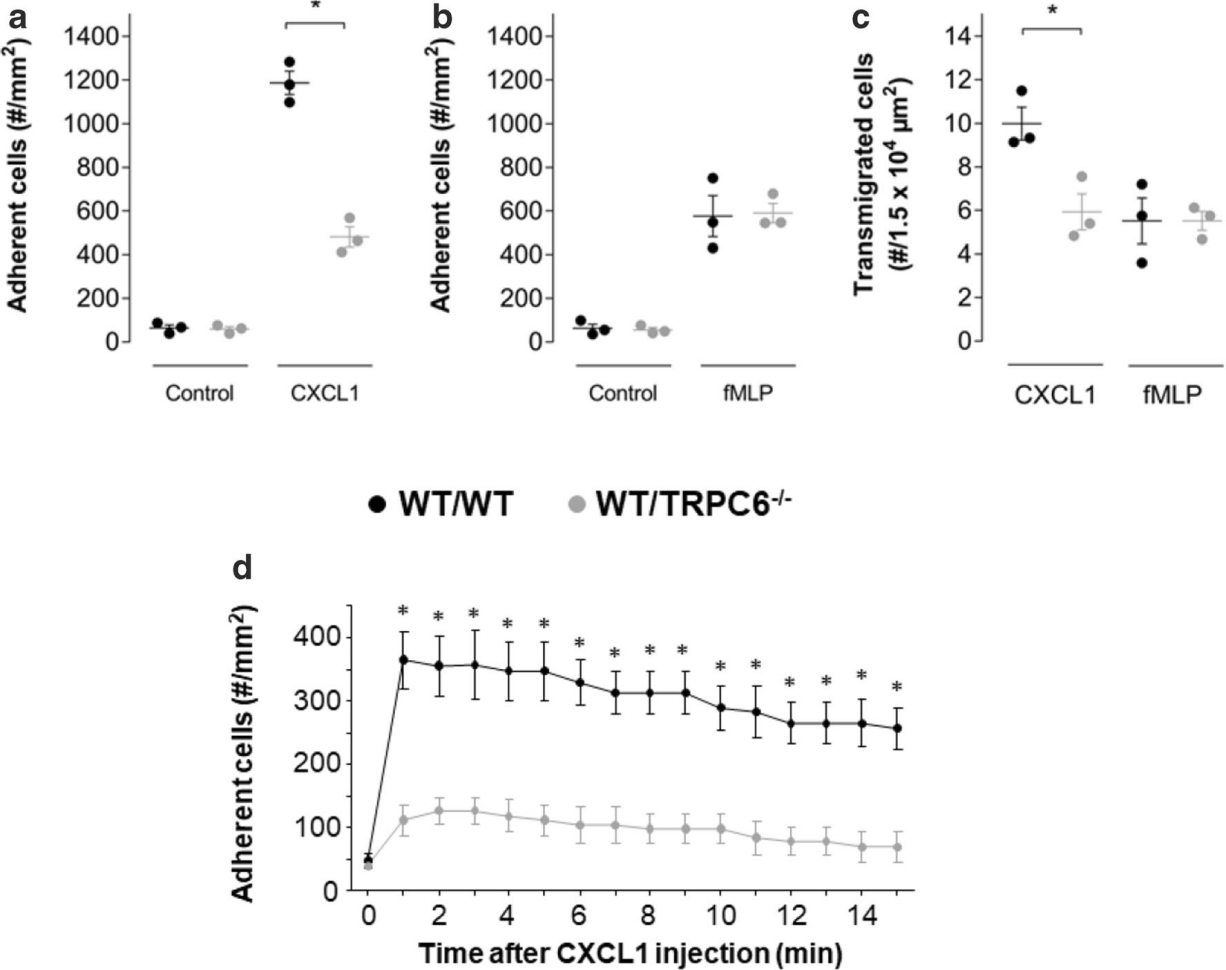
Inflammatory recruitment of neutrophils is impaired in WT/TRPC6^−/−^ mice. The number of arrested, tightly adherent (**a**, **b**) and transmigrated (**c**) leukocytes in postcapillary venules of the cremaster muscle of WT/WT and WT/TRPC6^−/−^ mice were analyzed by intravital microscopy. Arrest and transmigration of neutrophils were induced by superfusing the cremaster muscle with CXCL1 or fMLP containing Ringer’s solution for 2 h. (**d**) Intravascular injection of CXCL1 induces an immediate chemokine-induced leukocyte arrest in WT/WT mice but not in WT/TRPC6^−/−^ mice (*N* = 4 mice/group). The value at *t* = 0 reflects the control conditions prior to the injection of CXCL1. Values are reported as mean values ± SEM. *p < 0.05

Neutrophil recruitment in postcapillary venules of the cremaster muscle was investigated by intravital microscopy. Under control conditions, i. e., before superfusing CXCL1 or fMLP over the cremaster muscle, only few firmly adherent or transmigrated leukocytes were detected. There was no difference between the two genotypes. Before superfusing CXCL1 we counted 87 ± 14 (WT/WT) and 76 ± 11 (WT/TRPC6^−/−^) firmly adherent neutrophils per square millimeter (Fig. 2a). The control numbers of the fMLP group were 63 ± 18 and 41 ± 9 neutrophils per square millimeter (Fig. 2b), respectively. When the cremaster muscle was superfused for 2 h with CXCL1, the number of adherent cells rose ~ 15-fold in WT/WT mice. In WT/TRPC6^−/−^ mice it was clearly lower in comparison to WT/WT animals (decrease by ~ 60%) (Fig. 2a). Using fMLP, the number of firmly adherent cells rose ~ 11-fold, but there was no difference between WT/WT and WT/TRPC6^−/−^ mice (Fig. 2b). Similar results were observed for transmigrated cells. When superfusing CXCL1, the number of transmigrated cells was 41% lower in WT/TRPC6^−/−^ mice than in WT/WT mice. Stimulation with fMLP elicited no difference between WT/WT and WT/TRPC6^−/−^ mice (Fig. 2c).

In the next set of experiments, we tested the acute effect of an intravascular injection of CXCL1 on neutrophil arrest. The number of adherent neutrophils under control conditions is represented by the value at *t* = 0 min in Fig. 2d. Prior to the injection of CXCL1 or fMLP (corresponding to *t* = 0), the number of arrested and tightly adherent neutrophils amounted to 48 ± 10 and 40 ± 4 cells/mm^2^ in WT/WT and WT/TRPC6^−/−^ mice, respectively (Fig. 2d). Intravascular arrest of neutrophils remained almost unchanged in WT/TRPC6^−/−^ in comparison with WT/WT mice. In WT/WT mice, the number of firmly arrested neutrophils rose more than 6-fold (Fig. 2c). This indicates that TRPC6 channels play a prominent role in neutrophil adhesion on endothelial cells. TRPC6 channels are crucial for CXCL1-triggered adhesion.

### TRPC6 regulates Ca^2+^ signaling after initial selectin contact of neutrophils

**Fig. 3:**
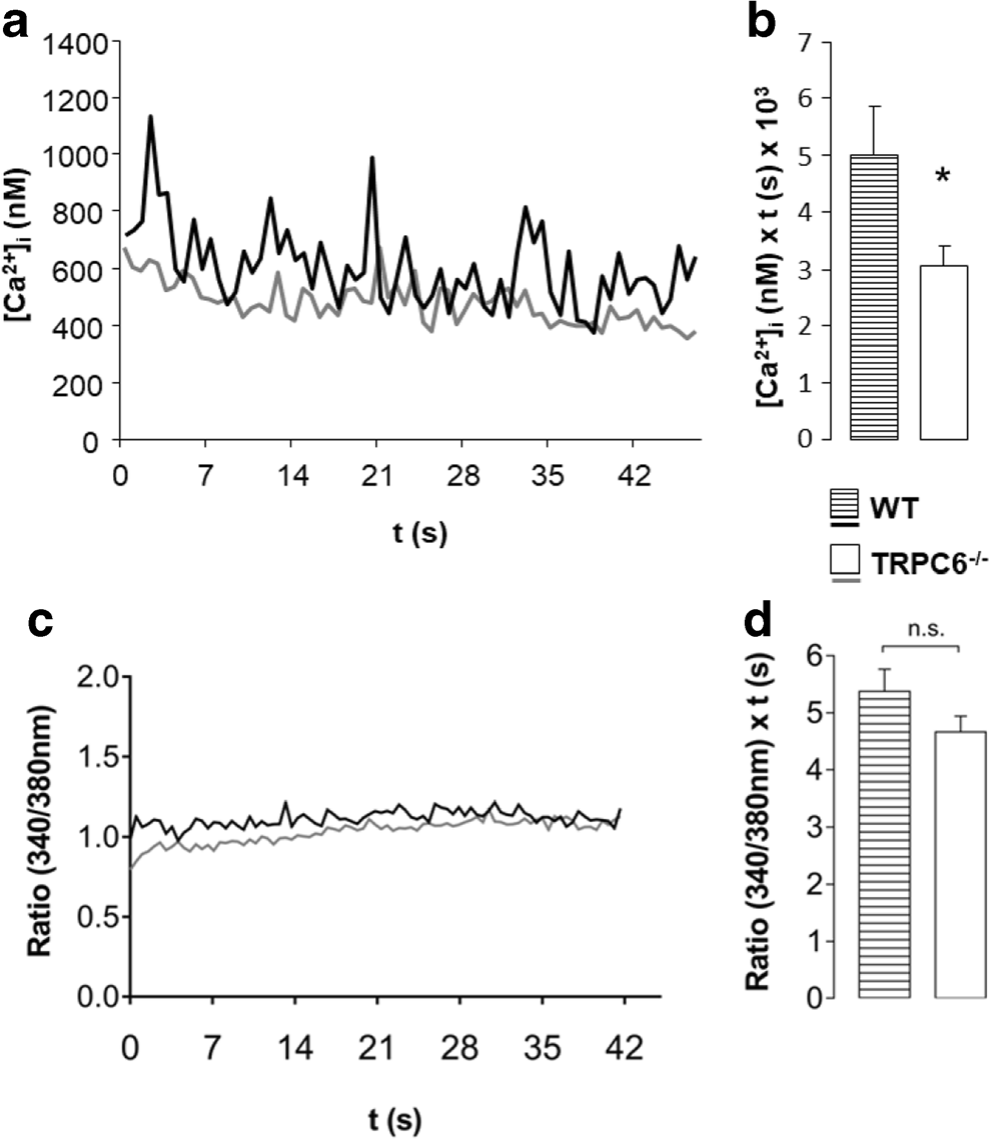
[Ca^2+^]_i_ concentration after initial selectin contact is decreased in TRPC6^−^/^−^ neutrophils. Mean [Ca^2+^]_i_ response of WT and TRPC6^−/−^ neutrophils after initial contact to a surface coated with E-selectin and CXCL1 (**a**) and corresponding integrals of the [Ca^2+^]_I_ for the first 5 s (**b**) (*n* ≥ 52 cells, *N* ≥ 3 mice). (**c**) Same experiment as in **a**, except that the surface is coated only with E-selectin and the corresponding integrals of the [Ca^2+^]_i_ for the first 5 s (**d**) (*n* ≥ 43 cells, *N* ≥ 3 mice). Values are reported as mean values ± SEM. *p < 0.05

The intracellular Ca^2+^ mobilization after initial contact of neutrophils with selectins was analyzed in flow chambers coated with E-selectin and CXCL1. During rolling and initial selectin contact, [Ca^2+^]_i_ increased up to 1100 nM in WT neutrophils, but only up to 700 nM in TRPC6^−/−^ cells (see Fig. 3a). [Ca^2+^]_i_ returned to baseline levels of 100–200 nM within a few minutes. We only analyzed the changes of [Ca^2+^]_i_ observed during the first 5 s after initial selectin contact in detail in order to avoid a confounding effect of integrin-mediated outside-in signaling in neutrophils that might have resulted from an interaction with the cell culture treated surface of the flow chambers. Using a similar microfluidic approach, it had been shown that an LFA1-induced increase of [Ca^2+^]_i_ occurs only after a lag period of more than 40 s [41]. The [Ca^2+^]_i_ integral was 39% smaller in TRPC6^−/−^ neutrophils than in WT cells (see Fig. 3b).

To distinguish between a CXCL1- or E-selectin-triggered increase in [Ca^2+^]_i,_ we repeated these experiments with flow chambers that were coated with E-selectin only. These experiments were complicated by the fact that the number of firmly adherent neutrophils was largely reduced and the vast majority of cells rolled out of the visual filed within a few seconds so that the measurements could not be calibrated. Consequently, [Ca^2+^]_i_ was assessed qualitatively in form of the ratio *F*_340_/*F*_380_. As evident from Fig. 3 c and d, neutrophils from WT and TRPC6^−/−^ mice behave alike. This is consistent with TRPC6 channels being required for the CXCL1-induced rise of [Ca^2+^]_i_.

### TRPC6 activates CXCL1-mediated adhesion of neutrophils

**Fig. 4:**
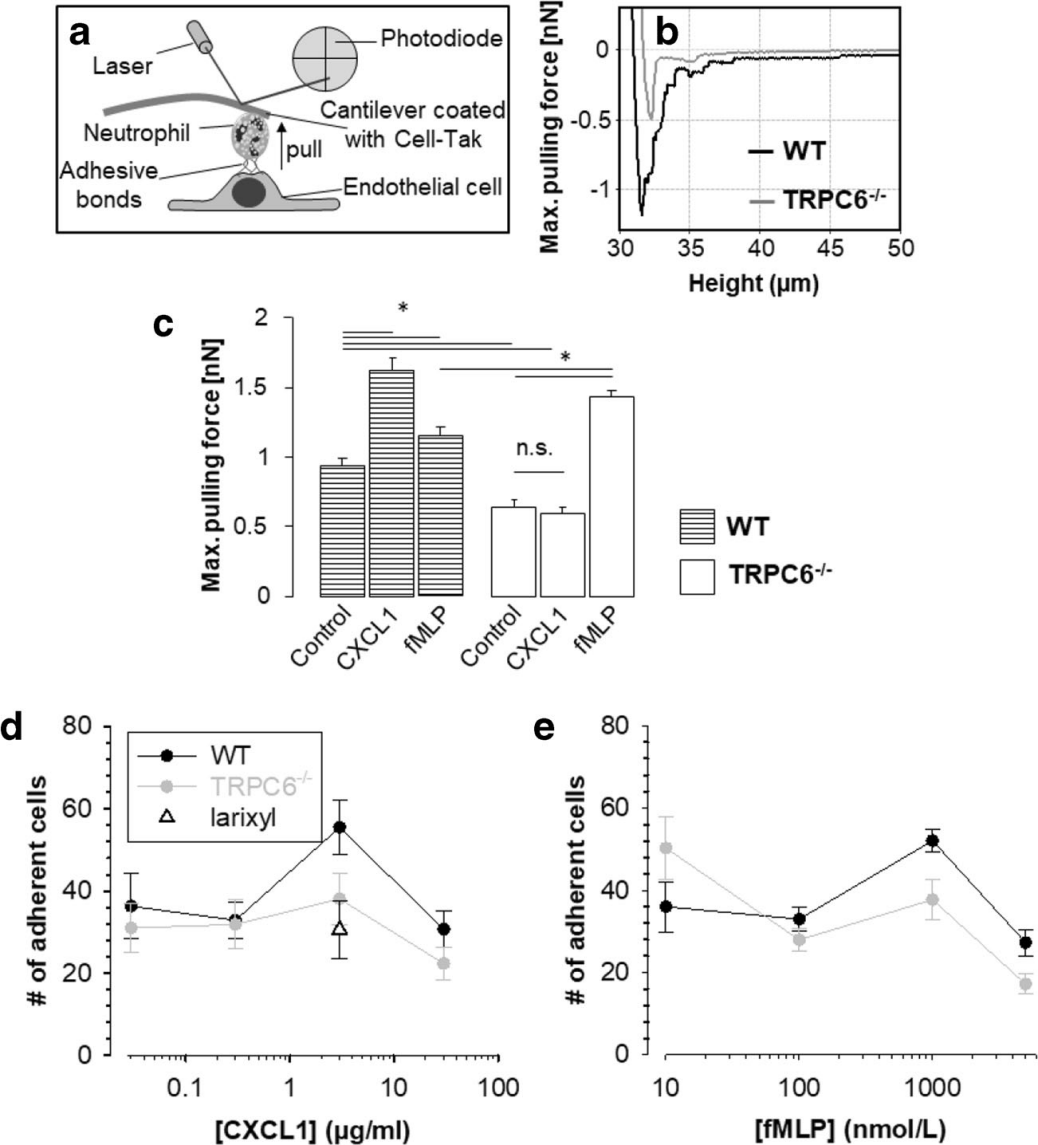
Neutrophil adhesion is decreased by loss of TRPC6. Experimental setup for single cell force spectroscopy (**a**), representative force-distance curves of neutrophils stimulated with CXCL1 and adhering to endothelial cells (**b**), and summary of force spectroscopy of neutrophils (**c**) (*n* ≥ 5 neutrophils from *N* ≥ 3 mice. Adhesion measurements were performed on 10 endothelial cells per neutrophil attached to the cantilever). (**d**) Summary of a microfluidic adhesion assay in which the number of CXCL1-stimulated WT and TRPC6^−/−^ neutrophils adhering to an E-selectin-coated flow chambers was quantified (larixyl: 5 μmol/L larixyl acetate; *N* = 5 mice for each genotype). **e** Summary of a microfluidic adhesion assay in which the number of fMLP-stimulated WT and TRPC6^−/−^ neutrophils adhering to an E-selectin coated flow chambers was quantified (neutrophils from *N* = 3 WT mice and *N* = 4 TRPC6^−/−^ mice). Values are reported as mean values ± SEM. *p < 0.05

Adhesion forces between neutrophils and endothelial cells were quantified with single-cell force spectroscopy using atomic force microscopy (Fig. 4a). To mimic the rapid time course of intravascular adhesion, neutrophils adhering to the AFM cantilever were placed onto an endothelial cell for only 1 s with a force of 1 nN. Then, the cantilever was lifted and maximum adhesion forces between endothelial cells and neutrophils were measured (Fig. 4b). Stimulation with both CXCL1 and fMLP increased adhesion forces in WT neutrophils (Fig. 4c). In TRPC6^−/−^ neutrophils, stimulation with CXCL1 had no effect, while fMLP could still elicit an increase in the adhesion forces to a level that was even 25% higher than in WT neutrophils. These data show that CXCL1-mediated adhesion is controlled by TRPC6 channels and that TRPC6^−/−^ neutrophils do not have a global defect in responding to chemoattractant stimulation. Moreover, they are in line with our findings described above showing the requirement of TRPC6 channels for CXCL1-triggered adhesion of neutrophils to postcapillary venules.

Experiments with single-cell force spectroscopy were complemented with a microfluidic adhesion assay with E-selectin-coated flow chambers in which CXCL1 and fMLP were tested at different concentrations (Fig. 4d, e). Maximum adhesion of neutrophils from both genotypes was achieved with 3 μg/μl CXCL1; lower and higher CXCL1 concentrations elicited less adhesion. However, at the optimal CXCL1 concentration there were ~ 50% more adherent WT neutrophils than adherent TRPC6^−/−^ neutrophils. When neutrophils were stimulated with fMLP, there was no significant difference between the two genotypes at any of the tested concentrations (Fig. 4e).

The role of TRPC6 channels in CXCL1-induced adhesion of neutrophils was further corroborated by performing in vitro adhesion assays in the absence and presence of the TRPC6 channel blocker larixyl acetate [42]. In the presence of 5 μmol/L larixyl acetate, CXCL1 (3 μg/μl) failed to increase the number of adherent WT neutrophils (Fig. 4d). It remained at the same level as observed for TRPC6^−/−^ neutrophils.

### CXCR2-mediated Rap1 activation depends on TRPC6 channels

**Fig. 5:**
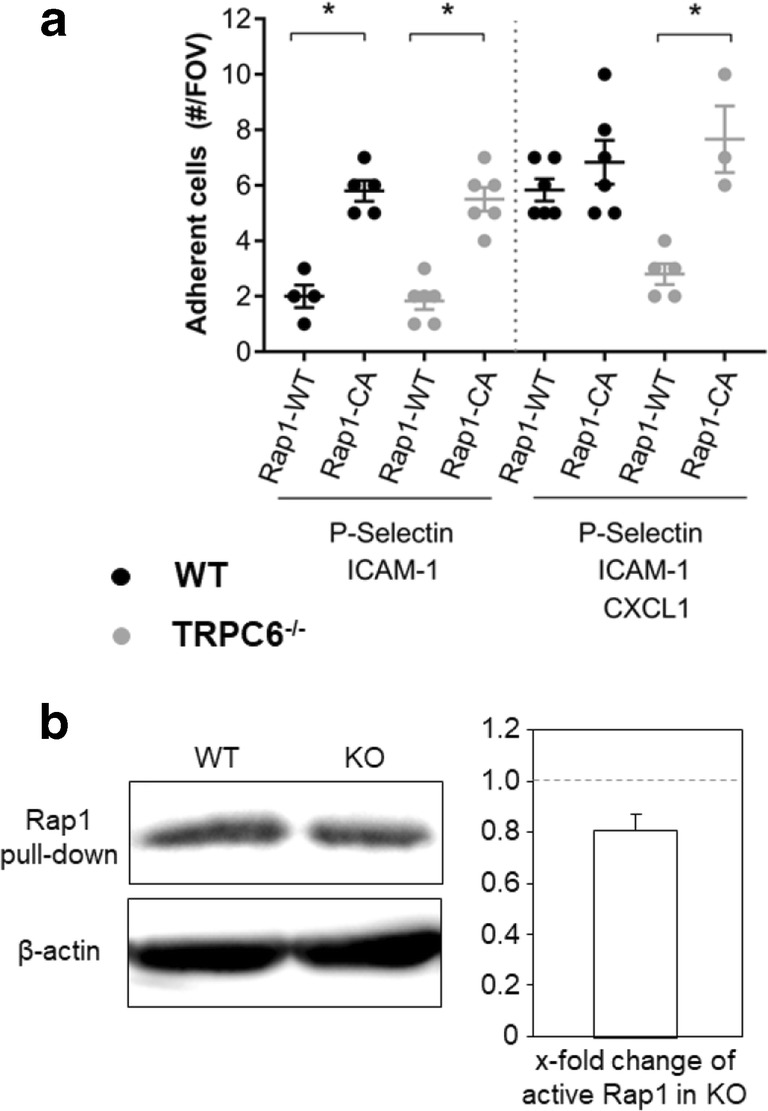
Chemoattractant-mediated Rap1 activation depends on TRPC6 channels. (**a**) Effect of WT or constitutive active Rap1 Tat-fusion mutants on neutrophil adhesion under flow ± CXCL1 stimulation (*n* ≥ 3 experiments from *N* ≥ 3 mice). Values are reported as mean values ± SEM. *p < 0.05. (**b**) Western blot analysis of active Rap1 after CXCL1 stimulation. For densiometric quantification of x-fold change in protein expression, equal loading was normalized to expression of β-actin. Values are reported as mean values ± SEM from *N* = 3 experiments with neutrophil lysates pooled from two mice for each experiment

The small GTPase Rap1 (Ras-related protein 1) has been shown to be involved in Ca^2+^ dependent integrin activation [26, 43]. Rap1-regulated adhesion of WT and TRPC6^−/−^ neutrophils was analyzed with TAT-fusion mutants of Rap1 in a microflow chamber assay (Fig. 5a). TAT-coupled Rap1-WT served as control, and a TAT-coupled constitutively active mutant of Rap1 (TAT-Rap1-CA) was used to activate integrin-mediated adhesion of the cells. In the absence of CXCL1, WT and TRPC6^−/−^ neutrophils treated with the Tat-Rap1-WT peptide adhered poorly in flow chambers coated with P-selectin/ICAM-1. The constitutively active mutant TAT-Rap1-CA elicited a robust increase in the number of adherent neutrophils of both genotypes. Similarly, the presence of the chemokine CXCL1 in the flow chambers induced a strong rise in the number of adherent WT neutrophils. Treatment of WT neutrophils with the mutant TAT-Rap1-CA had no additional effect. In contrast, CXCL1 failed to increase adhesion of TRPC6^−/−^ neutrophils. This defect could be rescued by pretreating TRPC6^−/−^ neutrophils with the constitutively active Tat-Rap1-CA peptide.

Rap1 activation was analyzed in Rap1 pull-down assays. After CXCL1 stimulation, the amount of active Rap1 protein was ~ 30% lower in TRPC6^−/−^ than in WT neutrophils (Fig. 5b). These findings suggest that TRPC6 channels regulate the CXCR2-mediated adhesion of neutrophils by activating Rap1. Accordingly, the adhesion defect of TRPC6^−/−^ neutrophils can be rescued by treating them with a constitutively active Rap1 protein.

Taken together, our results are consistent with the notion that Rap1 activation triggered downstream signaling leading to a conformational switch of β_2_-integrins to the high affinity conformation necessary for firm adhesion. The constitutively active Rap1 is able to overcome the functional defect of TRPC6^−/−^ neutrophils. Thus, these results provide indirect evidence for a link between TRPC6 channels and Rap1.

### ICAM-1 binding after CXCR2 activation is reduced in TRPC6^−/−^ neutrophils

**Fig. 6:**
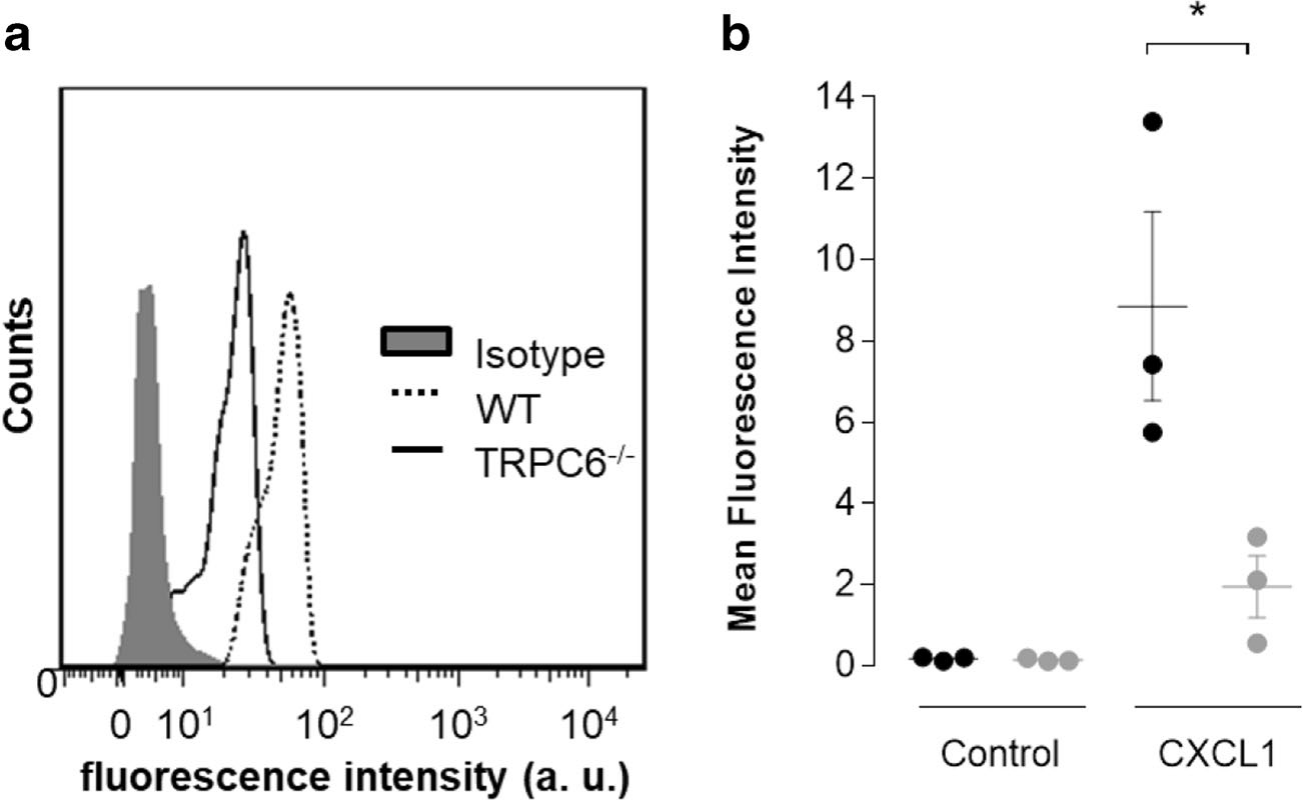
CXCR2-mediated ICAM-1 binding depends on TRPC6 channels. Flow cytometric measurement of ICAM-1 binding to neutrophils (*N* = 3 mice/group). **a** Representative histograms. For clarity reasons, we only depict the histograms for CXCL1-stimulated neutrophils. **b** Summary of ICAM-1-binding experiments. Values are reported as mean values ± SEM. *p < 0.05

Intravascular firm adhesion of neutrophils is mediated by the activated β_2_-integrin LFA-1 [6]. Integrin activation can be assessed by quantifying ICAM-1 binding by neutrophils. In order to determine LFA-1 dependent ICAM-1 binding, neutrophils were pretreated with a blocking Mac-1 antibody because Mac-1 is the only other binding partner of ICAM-1 on neutrophils [44]. ICAM-1 binding was measured under control conditions or after 3 min of CXCL1 stimulation by flow cytometry (Fig. 6). Under control conditions, the ICAM-1 binding was low, and no difference could be detected between WT and TRPC6^−/−^ neutrophils. After CXCL1 stimulation, ICAM-1 binding increased in both cell types. However, the rise was 4.6-fold higher in WT than in TRPC6^−/−^ neutrophils. The strong TRPC6-dependent increase in ICAM-1 binding can be attributed to LFA1 because Mac1 was functionally blocked in these experiments. These results lend further support to the involvement of TRPC6 channels in CXCR2-induced LFA-1 activation.

## Discussion

During an inflammation, chemoattractants trigger intracellular signaling cascades in neutrophils that usually involve intracellular Ca^2+^ transients. While the molecular identity of the underlying Ca^2+^-permeable channels has begun to be unraveled (reviewed in [16, 17]), their functional roles in the steps of the neutrophil recruitment cascade and their effector proteins have not yet been clarified in detail.

We previously demonstrated that TRPC6 channels are crucial for neutrophil chemotaxis in a collagen gel [12]. Similarly, eosinophil recruitment during an allergic airway response requires TRPC6 channels. The latter was hypothesized to be due to TRPC6 induced non-store operated Ca^2+^ influx and β_2_-integrin activation [45, 46]. On the other hand, deletion of TRPC6 channels confers renoprotection in a model of nephrosis. This is also associated with a reduced number of infiltrating monocytes and macrophages [47]. It was also shown that endothelial TRPC6 channels are needed for neutrophil recruitment [19]. In our previous study [12], we could not distinguish whether TRPC6 channels expressed in neutrophils or endothelial cells are needed for the recruitment to the peritoneal cavity because we used a global TRPC6^−/−^ mouse model. By employing chimeric mice with bone marrow cells from TRPC6^−/−^ mice, we can now clearly state that TRPC6 channels in neutrophils are essential for their CXCR2-dependent recruitment from the blood stream.

The present study shows that TRPC6 channels in neutrophils are essential for CXCR2-mediated transendothelial recruitment. This conclusion is based on the following key results: (1) firm intravascular adhesion and transmigration of neutrophils in vivo requires TRPC6 channels; (2) renal damage and neutrophil recruitment were diminished in the WT/TRPC6^−/−^ chimeras after a renal ischemia/reperfusion injury; (3) the increase of [Ca^2+^]_i_ after initial contact to an E-selectin and CXCL1-coated surface is partially dependent on TRPC6 channels; E-selectin coating alone elicits no TRPC6-dependent Ca^2+^ signals; (4) CXCR2-induced adhesion of neutrophils to E-selectin-coated surfaces and to endothelial cells requires TRPC6 channels; (5) Rap1-induced neutrophil adhesion and LFA1-mediated ICAM-1 binding depend on TRPC6 channels.

**Fig. 7:**
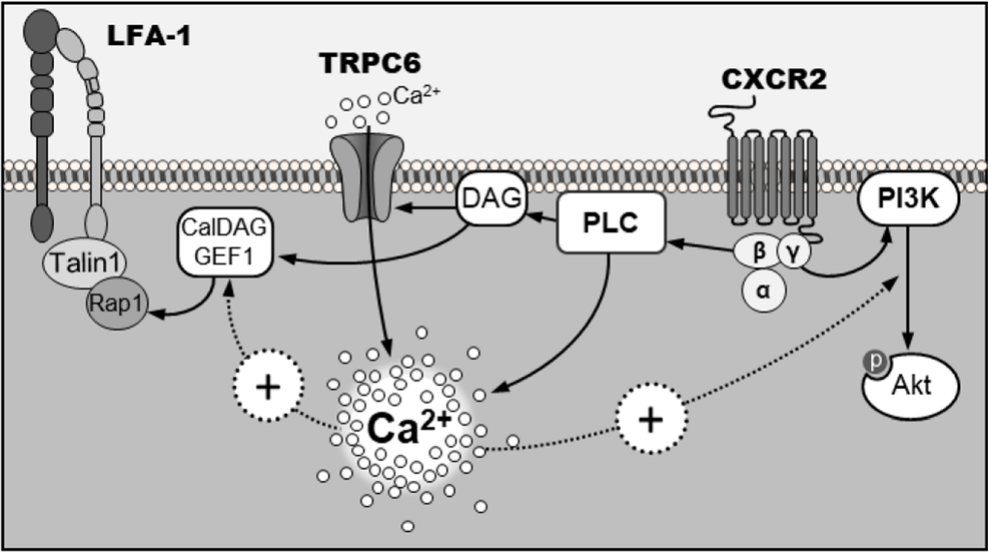
Integrin activation via CXCR2 and TRPC6. Chemokine binding of CXCR2 activates PLC triggering Ca^2+^ mobilization via store depletion and TRPC6 channels. This activates CalDAG-GEF1 which in turn activates Rap-1 mobilizing Talin1-β_2_ integrin association

In our previous study, we had demonstrated that TRPC6 deficiency leads to impaired Ca^2+^ signaling after CXCR2 stimulation of neutrophils [12]. This was recapitulated in the present study under more physiological conditions. The rise of [Ca^2+^]_i_ after initial selectin contact and CXCR2 activation was also significantly lower in TRPC6^−/−^ than in WT neutrophils. This TRPC6-dependent rise of [Ca^2+^]_i_ occurred much faster than the one involving integrin signaling [41]. The most likely explanation is that CXCR2 triggers the activation of TRPC6 via PLCβ-mediated DAG production [48]. In our view, this attenuation of Ca^2+^ transients in TRPC6^−/−^ neutrophils is likely to have implications for further downstream targets involved in the activation of integrins. CalDAG-GEF1 is activated by DAG and Ca^2+^, the latter with a K_D_ of 1.3 μmol/l [49]. This value is exactly in the range of the [Ca^2+^]_i_ elevations observed in rolling WT neutrophils in our experiments. In TRPC6^−/−^ neutrophils, [Ca^2+^]_i_ only rises to 700 nmol/L. We can therefore assume that CalDAG-GEF1 is not fully activated in TRPC6^−/−^ neutrophils. This could be an explanation for our findings that CXCR2-mediated Rap1 activation depends on TRPC6 channels because Rap1 is a downstream target of CalDAG-GEF1. Importantly and in line with our findings, Rap1 is involved in β_2_-integrin-mediated slow rolling of neutrophils [26, 43]. Thus, we propose that the TRPC6-mediated rise of [Ca^2+^]_i_ links the activation of CXCR2 indirectly to Rap1 stimulation (Fig. 7) which initiates the activation of LFA1 or Mac1 by its interaction with Talin1 and RIAM (Rap1-GTP-interacting adapter molecule) [50, 51]. The fact that the rise of [Ca^2+^]_i_ is not completely abolished in TRPC6^−/−^ neutrophils is also reflected by our results. Both the activation of Rap1 and the increase in ICAM-1 binding upon CXCL1 stimulation are attenuated in TRPC6^−/−^ neutrophils but not completely absent. Future studies need to address in a quantitative way whether (TRPC6-dependent) Rap1 activation and neutrophil adhesion are linearly correlated or whether there is a “threshold” of Rap1 activation beyond which neutrophil adhesion is maximal.

Our findings that altered Ca^2+^ signaling in TRPC6^−/−^ neutrophils leads to impaired neutrophil function is in line with other studies that also revealed a critical dependence of neutrophil behavior on Ca^2+^ signaling. Notably, both an increase and a decrease in the intracellular Ca^2+^ signals are accompanied by impaired neutrophil function. Increased Ca^2+^ signals are found in TRPC1^−/−^ [11] and in CF neutrophils [52]. The affected neutrophils have a defect in chemotaxis and ROS production as well as antimicrobial killing capacity, respectively. Intracellular Ca^2+^ signaling is attenuated in STIM1^−/−^ neutrophils and linked to impaired chemotaxis and infiltration of psoriatic lesions [53]. TRPV4^−/−^ neutrophils exhibit reduced Ca^2+^ transients following stimulation with PAF or 5,6-EET. Consequences are reduced ROS production, neutrophil adhesion, and chemotaxis [15].

Interestingly, neutrophils that are deficient for the actin-binding protein HS1 have the same complex phenotype as TRPC6^−/−^ neutrophils which we described in this and our previous paper [12, 54]: CXCL1-stimulated adhesion, transmigration, initial arrest, and recruitment into the inflamed peritoneal cavity are impaired. Moreover, HS1^−/−^ neutrophils share the same defect in chemotaxis with TRPC6^−/−^ neutrophils, while migration itself is also largely unaffected. Mechanistically, the phenotype of HS1^−/−^ neutrophils could be linked to a reduced Rap1 activation. In our view, these striking parallels lend support to the notion that TRPC6- as well as HS1-dependent and CXCL1-triggered signaling pathways utilize Rap1 as the same downstream effector protein.

We could show in a quantitative manner that chemoattractant receptor activation leads to doubling of adhesion forces of neutrophils adhering to endothelial cells. These findings are in good agreement with our dynamic assays using microfluidic devices. Nonetheless, single-cell spectroscopy provides detailed additional information. It allows the quantification of forces required for single unbinding events [55]. This makes the single-cell force spectroscopy a powerful tool to quantitatively analyze and compare adhesion characteristics of cells adhering to different surfaces.

In conclusion, our data show an important role for TRPC6 channels in transendothelial, CXCL1-triggered recruitment of neutrophils. TRPC6 channels are mediators of CXCL1-induced Ca^2+^ mobilization, which is essential for integrin activation during the first steps of neutrophil recruitment. Thereby, CXCR2-mediated activation of integrins is tightly regulated by TRPC6 channels. This study shows that TRPC6 channels could be interesting therapeutic targets for the pharmacological inhibition during inflammatory diseases involving CXCR2. In patients with acute kidney injury or in diseases like chronic obstructive pulmonary disease (COPD) which are based on the excessive CXCR2-driven recruitment of neutrophils [56], TRPC6 channel inhibition could represent a mild therapeutic approach with fewer side effects that improves patient outcome without completely downregulating the neutrophil function. Similarly, the CXCR2-dependent recruitment of neutrophil granulocytes to the stroma of pancreatic cancer where they elicit high immunosuppressive activity and drive disease progression [57] could potentially be targeted by TRPC6 blockers.
